# Peri-operative pulse oximetry in low-income countries: a cost–effectiveness analysis

**DOI:** 10.2471/BLT.14.137315

**Published:** 2014-09-24

**Authors:** Samantha L Burn, Peter J Chilton, Atul A Gawande, Richard J Lilford

**Affiliations:** a School of Health and Population Sciences, University of Birmingham, Edgbaston, Birmingham B15 2TT, England.; b Ariadne Labs at Brigham and Women's Hospital and the Harvard School of Public Health, Boston, United States of America.; c Warwick Medical School, University of Warwick, Coventry, England.

## Abstract

**Objective:**

To evaluate the cost–effectiveness of pulse oximetry – compared with no peri-operative monitoring – during surgery in low-income countries.

**Methods:**

We considered the use of tabletop and portable, hand-held pulse oximeters among patients of any age undergoing major surgery in low-income countries. From earlier studies we obtained baseline mortality and the effectiveness of pulse oximeters to reduce mortality. We considered the direct costs of purchasing and maintaining pulse oximeters as well as the cost of supplementary oxygen used to treat hypoxic episodes identified by oximetry. Health benefits were measured in disability-adjusted life-years (DALYs) averted and benefits and costs were both discounted at 3% per year. We used recommended cost–effectiveness thresholds – both absolute and relative to gross domestic product (GDP) per capita – to assess if pulse oximetry is a cost–effective health intervention. To test the robustness of our results we performed sensitivity analyses.

**Findings:**

In 2013 prices, tabletop and hand-held oximeters were found to have annual costs of 310 and 95 United States dollars (US$), respectively. Assuming the two types of oximeter have identical effectiveness, a single oximeter used for 22 procedures per week averted 0.83 DALYs per annum. The tabletop and hand-held oximeters cost US$ 374 and US$ 115 per DALY averted, respectively. For any country with a GDP per capita above US$ 677 the hand-held oximeter was found to be cost–effective if it prevented just 1.7% of anaesthetic-related deaths or 0.3% of peri-operative mortality.

**Conclusion:**

Pulse oximetry is a cost–effective intervention for low-income settings.

## Résumé

**Objectif:**

Évaluer la rentabilité de l'oxymétrie de pouls par rapport à l'absence de surveillance périopératoire lors d'une chirurgie dans les pays à revenu faible.

**Méthodes:**

Nous avons considéré l'utilisation d'oxymètres de pouls à poser et d'oxymètres de pouls portatifs chez des patients de tous âges ayant subi une opération chirurgicale importante dans des pays à revenu faible. À partir d'études antérieures, nous avons obtenu la mortalité de référence et l'efficacité des oxymètres de pouls pour réduire la mortalité. Nous avons considéré les coûts directs de l'achat et de l'entretien des oxymètres de pouls, ainsi que le coût de l'oxygène supplémentaire utilisé pour traiter les épisodes hypoxiques identifiés par oxymétrie. Les avantages pour la santé ont été mesurés en espérance de vie corrigée de l'incapacité (EVCI) évitée, et les avantages et les coûts ont été actualisés à 3% par an. Nous avons utilisé les seuils de rentabilité recommandés – à la fois de manière absolue et relative par rapport au produit intérieur brut (PIB) par habitant – afin d'évaluer si l'oxymétrie de pouls était une intervention de santé rentable. Pour tester la solidité de nos résultats, nous avons effectué des analyses de sensibilité.

**Résultats:**

Avec les prix de 2013, les oxymètres à poser et les oxymètres portatifs présentaient des coûts annuels de 310 et 95 dollars, respectivement. En supposant que les deux types d'oxymètre ont une efficacité identique, un seul oxymètre utilisé pour 22 interventions par semaine permettait d'éviter 0,83 EVCI par an. Les oxymètres à poser et les oxymètres portatifs coûtaient 374 $ et 115 $ par EVCI évitée, respectivement. Pour tous les pays avec un PIB par habitant supérieur à 677 $, l'oxymètre portatif s'est avéré rentable s'il évitait seulement 1,7% des décès liés à l'anesthésie ou 0,3% de mortalité périopératoire.

**Conclusion:**

L'oxymétrie de pouls est une intervention rentable pour les pays à faible revenu.

## Resumen

**Objetivo:**

Evaluar la rentabilidad de la oximetría de pulso en comparación con la ausencia de vigilancia perioperatoria durante la cirugía en países de ingresos bajos.

**Métodos:**

Se tuvieron en cuenta oxímetros de pulso manuales, tanto de mesa como portátiles, entre los pacientes de todas las edades sometidos a una intervención quirúrgica importante en países de ingresos bajos. A partir de los estudios anteriores se obtuvo la mortalidad inicial y la eficacia de los oxímetros de pulso para reducir la mortalidad. Se consideraron los costes directos de la adquisición y del mantenimiento de los oxímetros de pulso, así como el coste del oxígeno complementario que se utiliza para tratar los episodios de hipoxia identificados mediante la oximetría. Se midieron los beneficios para la salud en años de vida con discapacidad (AVAD) evitados, mientras que los beneficios y los costes se descontaron al 3% por año. Utilizamos los umbrales de rentabilidad recomendados, tanto absolutos como relativos, respecto al producto interno bruto (PIB) per cápita para evaluar si la oximetría de pulso es una intervención de salud rentable. Por último, se realizó un análisis de sensibilidad para poner a prueba la solidez de los resultados.

**Resultados:**

En 2013, se halló que los precios de los oxímetros de mesa y portátiles suponían unos costes anuales de 310 y 95 dólares estadounidenses (US$), respectivamente. En el supuesto de que ambos tipos de oxímetro tengan la misma eficacia, el uso de un único oxímetro para 22 procedimientos por semana evitó 0,83 AVAD por año. Los oxímetros de mesa y manuales cuestan 374 US$ y 115 US$ por AVAD evitado, respectivamente. Para cualquier país con un PIB per cápita superior a 677 US$ el oxímetro manual resultó ser rentable con tan solo impedir un 1,7% de las muertes relacionadas con la anestesia o el 0,3% de la mortalidad perioperatoria.

**Conclusión:**

La oximetría de pulso es una intervención rentable para entornos de ingresos bajos.

## ملخص

**الغرض:**

تقدير مردودية قياس التأكسج النبضي - مقارنة بعدم الرصد في الفترة المحيطة بالعملية - أثناء الجراحة في البلدان المنخفضة الدخل.

**الطريقة:**

قمنا بدراسة استخدام أجهزة قياس التأكسج النبضي التي توضع على أسطح الطاولات والقابلة للنقل والمحمولة باليد بين المرضى الذين ينتمون إلى أي فئة عمرية وخضعوا لجراحة كبرى في البلدان المنخفضة الدخل. وحصلنا من الدراسات السابقة على معدل الوفيات عند خط الأساس وفعالية أجهزة قياس التأكسج النبضي في تقليل معدل الوفيات. ودرسنا التكاليف المباشرة لشراء أجهزة قياس التأكسج النبضي وصيانتها بالإضافة إلى تكلفة الأوكسجين التكميلي الذي يستخدم لعلاج نوبات نقص الأوكسجين التي يحددها قياس التأكسج. وتم قياس الفوائد الصحية بسنوات العمر المصححة باحتساب مدد العجز التي تم تفاديها والفوائد وتم خفض التكاليف في كل منها بنسبة 3 % سنوياً. واستخدمنا عتبات المردودية الموصى بها - المطلقة والنسبية للناتج الإجمالي المحلي للفرد - لتقييم ما إذا كان قياس التأكسج النبضي تدخلاً صحياً ذا مردودية. ولاختبار قوة نتائجنا، نفذنا تحليلات الحساسية.

**النتائج:**

تبين وفق أسعار 2013 أن التكاليف السنوية لأجهزة قياس التأكسج التي توضع على أسطح الطاولات والمحمولة باليد تبلغ 310 و95 دولاراً أمريكياً، على التوالي. وبافتراض تطابق المردودية لنوعي أجهزة قياس التأكسج، يؤدي جهاز قياس التأكسج الواحد الذي يستخدم في 22 عملية أسبوعياً إلى تفادي 0.83 سنة من سنوات العمر المصححة باحتساب مدد العجز سنوياً. وتبلغ تكلفة أجهزة قياس التأكسج التي توضع على أسطح الطاولات والمحمولة باليد 374 دولاراً أمريكياً و115 دولاراً أمريكياً لكل سنة تم تفاديها من سنوات العمر المصححة باحتساب مدد العجز، على التوالي. وبالنسبة لأي بلد يزيد الناتج الإجمالي المحلي للفرد فيه عن 677 دولاراً أمريكياً، تبين أن جهاز قياس التأكسج المحمول باليد يكون ذا مردودية في حالة توقي 1.7 % فقط من حالات الوفاة ذات الصلة بالتخدير أو 0.3 % من معدل الوفيات في الفترة المحيطة بالعملية.

**الاستنتاج:**

يعتبر قياس التأكسج النبضي تدخلاً ذا مردودية في المناطق منخفضة الدخل.

## 摘要

**目的:**

评估低收入国家中手术过程脉搏血氧测量与无围术期监测相比的成本效益。

**方法:**

我们考虑低收入国家在任何年龄病人接受大手术中对台式和便携式手持脉搏血氧测量仪的使用情况。我们从早期的研究中得到基线死亡率和脉搏血氧测量仪降低死亡率的有效性。我们考虑购买和维护脉搏血氧测量仪的直接成本以及用于治疗由血氧测量确定的间歇低氧发作的补充氧气的成本。以避免的伤残调整生命年(DALY)测量健康益处,收益和成本每年都折减3%。我们使用推荐的成本效益阈值(绝对值以及与人均国内生产总值(GDP)对比的相对值)评估脉搏血氧测量仪是否是具有成本效益的健康干预。我们执行敏感性分析来测试结果的稳健性。

**结果:**

以2013年的价格计算,台式和手持血氧测量仪年度成本分别为310和95美元。假设这两种类型的血氧测量仪具有相同的效果,单个血氧测量仪每周用于22台手术过程,每年可避免0.83个DALY。台式和手持血氧测量仪避免一个DALY的成本分别为374和115美元。研究发现,对于任何人均国内生产总值超过677美元的国家,手持血氧测量仪仅避免1.7%的麻醉相关死亡或0.3%的围手术期死亡率就具有成本效益。

**结论:**

低收入的环境中,脉搏血氧测量是一种经济有效的干预措施。

## Резюме

**Цель:**

Оценить экономическую эффективность пульсоксиметрии — в сравнении с отсутствием периоперационного мониторинга — при проведении операций в странах с низким уровнем доходов.

**Методы:**

Было рассмотрено использование как настольных, так и портативных ручных пульсоксиметров среди пациентов любого возраста, перенесших обширное опреативное вмешательство, в странах с низким уровнем доходов. Данные об исходном уровне смертности и эффективности пульсоксиметров для снижения смертности были получены из предыдущих исследований. Учитывались прямые затраты на приобретение и обслуживание пульсоксиметров, а также стоимость дополнительного кислорода, используемого для устранения эпизодов гипоксии, выявленных с помощью оксиметрии. Полезность для здоровья определялась спасенными годами жизни, скорректированными на инвалидность (индекс DALY), при этом полученные значения полезности и затрат снижались на 3% в год. Были использованы рекомендованные пороговые значения показателя «затраты-эффективность» — как абсолютные, так и относительные, с учетом валового внутреннего продукта (ВВП) на душу населения — для оценки того, является ли пульсоксиметрия экономически эффективной лечебной мерой. Чтобы проверить надежность полученных результатов, был проведен анализ чувствительности.

**Результаты:**

Было установлено, что в ценах 2013 года годовые затраты на использование настольных и ручных оксиметров составили 310 и 95 долларов США соответственно. Если предположить, что два типа оксиметров имеют одинаковую эффективность, то один оксиметр, применяемый для 22 процедур в неделю, спас 0,83 DALY за год. Стоимость настольного и ручного оксиметра на спасенный DALY составила 374 и 115 долларов США соответственно. Для любой страны с ВВП на душу населения свыше 677 долларов США использование ручного оксиметра оказалось экономически эффективным, если он позволял предотвращать как минимум 1,7% анестезиологических или 0,3% периоперационных смертей.

**Вывод:**

Пульсоксиметрия является экономически эффективной лечебной мерой для стран с низким уровнем доходов.

## Introduction

The pulse oximeter is a non-invasive medical device that monitors oxygen saturation and pulsation. When used continuously during surgery, it can provide early warning of hypoxia, hypovolaemia and impending cardiac arrest. Since oximetry can warn of problems such as misplaced endotracheal tubes – which can readily be rectified – the World Federation of Societies of Anaesthesiologists recommends its routine use for every patient undergoing anaesthesia in the world.^1^^,^^2^ The World Health Organization (WHO) includes pulse oximetry as a component of its Surgical Safety Checklist, which is recommended for use in every operating theatre.^1^ However, it has recently been estimated that pulse oximetry is unavailable in 51–70% of operating theatres in low-income countries,^3^ partly because of the high purchase cost of a standard commercial tabletop pulse oximeter – approximately 1000 United States dollars (US$).^4^ The Lifebox oximetry project, which currently operates alongside the WHO Safe Surgery Saves Lives initiative, provides a hand-held pulse oximeter for low- and middle-income countries that costs US$ 250.^4^ However, even this smaller sum is a considerable investment for resource-constrained settings. Furthermore, because no evidence of the cost–effectiveness of pulse oximetry for peri-operative monitoring in low-income countries has yet been published, it is not clear how oximetry should be prioritized among the many cost–effective interventions available.^5^ In this paper, we conducted a cost–effectiveness analysis of pulse oximetry – compared with no peri-operative monitoring – for patients undergoing surgery in low-income countries. This study is based on a synthesis of data from previously published studies from a large number of different countries. While the group of low-income countries is heterogeneous, the analysis presented here is readily adaptable to specific national contexts.

## Methods

We investigated the equivalent annual costs of purchasing and maintaining pulse oximeters, as well as the costs of increased oxygen flow used to treat any hypoxic episodes identified by oximetry. We took a health services perspective. In quantifying the health benefits of peri-operative oximetry, we considered the number of disability-adjusted life-years (DALYs) averted by using pulse oximetry to reduce the incidence of fatal intra-operative hypoxic episodes. We ignored non-fatal cases of hypoxic brain injury. Base case analysis was conducted using version 3.0.1 of the R software package (R Foundation for Statistical Computing, Vienna, Austria) and Microsoft Excel 2010 (Microsoft, Redmond, USA) and sensitivity analysis using the TreeAge Pro 2013 software package (TreeAge Software Inc., Boston, USA).

### Costs

We only considered oximeters that met the IEC 60601–1, ISO 9919:2005 or ISO 80601–2–61:2011 international standards for safety and performance. There are two main types of stand-alone pulse oximeter designed for peri-operative use: the standard commercial tabletop oximeter and a less expensive hand-held device with similar functionality but a more portable and durable design and a rechargeable battery.^6^ The oximeter distributed by the Lifebox charity is of the second type. 

Costs are given in 2013 prices and discounted at 3% per year – as recommended in version two of the Disease Control Priorities in Developing Countries^5^ and the WHO-CHOICE^7^^,^^8^ guidelines for evaluation of the cost–effectiveness of health interventions in developing countries. Domestic taxes were excluded. We assumed that no extra operations would be carried out as a result of introducing oximetry and that no extra clinical staff time would be required. We included supplementary oxygen^9^ resulting from an increase in the incidence of detected hypoxia^10^ when oximetry is used.

### Health benefits

Most of the published data on the performance of oximeters relate to tabletop oximeters. However, the low-cost hand-held oximeter distributed by Lifebox has recently been found to perform as well as tabletop oximeters that have been produced by major manufacturers, made commercially available in the United States of America.^11^ For our analysis, we therefore assumed that the effectiveness of the hand-held devices in averting peri-operative death was identical to that of the tabletop devices.

Lifebox has found that the oximeters it distributes can be used for 25–30 surgeries per week.^12^ For our analysis, we assumed that each of the oximeters we investigated was used at about 80% of these frequencies^8^ – i.e. in 22 procedures per week.

#### Baseline mortality

We did a systematic search to identify systematic reviews of anaesthetic-related and total peri-operative mortality including studies from low-income countries published between 1 January 1990 and 31 December 2012. The search terms used included variants of anaesthetic, surgery, operation, intraoperative, peri-operative, peri-surgical, death, mortality and survival (Box 1). We searched the following databases: MEDLINE via OvidSP, EMBASE via OvidSP, Scopus, the Cochrane Database of Systematic Reviews, the Database of Abstracts of Reviews of Effects, the Health Technology Assessment Database and the International Prospective Register of Systematic Reviews. We used the Centre for Reviews and Dissemination filter (strategy 2.1) to identify systematic reviews.^13^ No language restrictions were applied.

Box 1 **Search strategy for key parameters published in systematic reviews**

**Baseline anaesthetic-related mortality**

1. Surgery[Mesh] OR surg* OR operat* OR perioperat* OR peri-operat* OR intraoperat* OR intra-operat* OR “in theatre”

2. Anesthesia[Mesh] OR anesth* OR anaesth* OR peri-anesth* OR perianesth* OR post-anesth* OR post-anaesth*

3. Death OR mortalit* OR morbidit* OR survival*

4. (#1 OR #2) AND #3

**Effectiveness of pulse oximetry in reducing hypoxic episodes and/or peri-operative mortality**

1. Surgery[Mesh] OR surg* OR operat* OR perioperat* OR peri-operat* OR intraoperat* OR intra-operat* OR “in theatre”

2. Oximet* OR oxymet*

3. Death OR mortalit* OR morbidit* OR survival*

4. Anoxia[Mesh] OR anox* OR hypox*

5. (#1 AND #2) AND (#3 OR #4)

Two systematic reviews of anaesthetic-related and total peri-operative mortality that included studies from low-income countries were identified.^14^^,^^15^ Since it was the more recent of the two reviews and included a formal meta-analysis, we used the study by Bainbridge et al.^14^ to parameterize our cost–effectiveness estimates. This study found that total peri-operative mortality in low-income countries – i.e. countries with a human development index below 0.8 – was 2445 deaths per million procedures.^14^ They also found that, in low-income countries, problems in the administration of anaesthesia – e.g. oesophageal intubation or kinking of the endotracheal tube – were the sole or contributing cause of 467 deaths per million procedures. We took 467 deaths per million procedures as our baseline for deaths that were potentially preventable by oximetry. However, this may be a conservative estimate, since oximetry could also provide an early warning of a deterioration in the patient’s underlying condition that was unrelated to anaesthesia. Also, as oximetry may have been used in one or more of the studies investigated by Bainbridge et al., 467 deaths per million procedures may represent an underestimate of mortality in the absence of oximetry.

For robustness, we replicated the search procedure used by Bainbridge et al.^14^ to identify any recent studies of relevance that had been published on or before 30 December 2012. We found no new studies relating to low-income countries. We also examined the six studies that were excluded by Bainbridge et al. because of small sample size^16^^,^^17^ or because they pertained exclusively to one clinical area.^18^^–^^21^ Two of these studies contained estimates of anaesthetic-related avoidable mortality for a general population, which were 1985 and 7500 deaths per million procedures. In our sensitivity analysis, we therefore considered values of anaesthetic-related avoidable mortality that varied from 253 deaths per million procedures – i.e. the lowest estimate from the studies investigated by Bainbridge et al.^14^ – to 7500 deaths per million procedures.

#### Effectiveness in reducing mortality

The available data on the effectiveness of pulse oximetry come from observational studies – e.g. before-and-after studies or critical incident reports – and randomized controlled trials. In this context, such studies and trials are imperfect. Since peri-operative deaths are extremely rare, none of the relevant randomized controlled trials is adequately powered to detect the effects of oximetry on the probability of such deaths.^10^^,^^22^ The relevant observational studies do not allow cause–effect statements to be made with confidence since such studies are confounded by temporal changes that are unrelated to oximetry.^23^^,^^24^

We did a systematic search to identify systematic reviews of the effectiveness of oximetry in preventing hypoxia and peri-operative death published between 1 January 1990 and 31 December 2012 (Box 1). We searched the same set of databases as for baseline anaesthetic-related mortality. We identified one systematic review of randomized controlled trials of the effectiveness of pulse oximetry, in which the authors concluded that pulse oximetry reduces the incidence of hypoxaemia by 33–67% but appears to have no statistically significant effect on mortality.^22^ To check the robustness of this result, we reviewed the studies that were excluded because they were not randomized.^22^ These excluded studies^25^^–^^27^ that indicated a similar oximetry-attributable decline in hypoxaemia to that observed in the included studies. Other observational data indicate that anaesthetic-related mortality in high-income countries has declined by 64% since the 1980s, as various monitoring standards, including pulse oximetry, have been widely implemented.^14^^,^^28^^,^^29^

While much of the evidence assembled relates to high-income countries, the results of a before-and-after study conducted in the Republic of Moldova indicated that the introduction of pulse oximetry – along with the entire WHO Surgical Safety Checklist – reduced the number of hypoxaemic episodes lasting at least two minutes by 44%.^30^ Another before-and-after study found that introduction of the checklist led to a 60% reduction in total peri-operative mortality over four study sites in low-income countries.^31^ However, the checklist contains several other items known to be associated with improved safety outcomes, so these reductions are probably not attributable to oximetry alone.

We selected 50% as the upper plausible limit for effectiveness of oximetry in reducing anaesthetic-related deaths, since this is the figure obtained using the surrogate outcome of hypoxic episodes in the randomized control trials described above.^10^^,^^22^ This a highly optimistic value, since surrogate outcomes are notorious for overestimating clinical benefit.^32^^–^^34^ We therefore used 50% as an upper bound for effectiveness. For a lower bound we selected a 2% improvement in anaesthetic-related mortality, to represent a very pessimistic estimate given the randomized control trials and observational evidence cited above. For the base case we used effectiveness of 10%, founded on the nature of the available evidence and based on discussion with our advisors. Since there is considerable uncertainty surrounding these values, we conducted extensive sensitivity analysis.

#### Disability-adjusted life-years averted

Health benefits were measured in DALYs averted, with uniform age-weighting and discounting at 3% per annum. DALYs were calculated using actual life expectancy rather than life expectancy for a hypothetical reference group.^5^ Using pooled health-adjusted life expectancy tables for the Eastern Sub-Sahara Global Burden of Disease region^35^ and a probability density function of the ages of patients undergoing major surgery in Mozambique, Uganda and the United Republic of Tanzania,^36^ we calculated that 15.5 DALYs are averted per anaesthetic-related death avoided.

We assumed that the sex distribution of patients was the same as that of the relevant national population. We also assumed that, in all cases of averted death, a patient’s health-adjusted life expectancy did not differ from that of the general population and that the benefits of pulse oximetry were too small to alter overall national life expectancies.

### Cost–effectiveness thresholds

We used two common types of cost–effectiveness thresholds for health interventions in low-income countries:^37^ the absolute thresholds used by the World Bank in the *World development report 1993*^38^ and the thresholds – defined relative to the corresponding gross domestic product (GDP) per capita – used by WHO-CHOICE.^7^ According to the *World development report 1993*, interventions that, in 1993, cost no more than US$ 25 and US$ 150 per DALY averted could be considered highly attractive and attractive, respectively. Assuming 3% inflation per year, the corresponding thresholds for the year 2013 would be US$ 45 and US$ 271. WHO-CHOICE considered interventions that, per DALY averted, cost no more than one and three times the relevant GDP per capita to be very cost–effective and cost–effective, respectively.^7^ For the group of low-income countries as a whole, US$ 677 and US$ 2031 are one and three times the 2013 GDP per capita, respectively.^39^

## Results

Our cost and cost–effectiveness estimates are summarized in Table 1 and Table 2, respectively. In the base case – comparing each type of oximeter with no monitoring of oxygen saturation and assuming both the tabletop and hand-held pulse oximeters reduce anaesthetic-related mortality by 10% – the costs per DALY averted were US$ 374 for the tabletop pulse oximeter and US$ 115 for the hand-held oximeter. Since we assume in this analysis that the effectiveness of the two types of oximeter is identical and the hand-held oximeter is less costly, the hand-held oximeter dominates the tabletop oximeter. The cost–effectiveness of the hand-held oximeter fell below the very cost–effective threshold of one times the GDP per capita for low-income countries.

**Table 1:** Costs of purchasing, maintaining and repairing pulse oximeters

Parameter	Point estimate (range)^a^		Data source(s) and assumptions^a^
	Commercial tabletop device	Hand-held device	
Cost of purchase, shipping and internal transport, US$	1065 (600–3000)	250 (250–280)	Lifebox product information^4^
Life-span, years	6 (4–8)	8 (6–10)	Expert opinion
Annuitized maintenance costs, including those for replacement probes and batteries, US$^b^	34 (17–85)	18 (15–31)	Probes for tabletop device replaced every 2 (1–3) years at a cost of US$ 100.^40^^,c^ Probes for hand-held device replaced every 2 (1–3) years at a cost of US$ 25^4^ and batteries for hand-held device replaced every year at a cost of US$ 10^4^
Annuitized repair costs, US$	45 (30–60)	6 (2–13)	15% (10–20%) chance of breakage of a tabletop device each year, at a total cost per year of US$ 355 including shipping.^40^^,c^ 5% (1–10%) chance of breakage of hand-held device each year, at a total cost per year of US$ 65 during the first 2 years – when the device is under warranty – and US$ 138 thereafter^4^
Annual cost of treating additional hypoxic episodes identified by pulse oximetry, US$	35 (17–52)	35 (17–52)	Incidence of hypoxia is 7.9% with pulse oximetry and 0.4% without oximetry.^10^ If hypoxia is detected, oxygen flow increased by 8 litres/min for 10 minutes,^c^ at a cost of US$ 0.40 per additional hypoxic episode detected^9^
Total equivalent annual cost, US$	310	95	Authors’ calculations. Uncertainty explored in sensitivity analysis

US$: United States dollars.

^a^ All costs are shown adjusted to 2013 values, assuming 3% inflation per year.

^b^ Excludes share of general overhead costs attributable to use of pulse oximetry – i.e. costs of cleaning and electricity.

^c^ Value partly or entirely based on expert opinion.

**Table 2:** Effectiveness and cost–effectiveness of pulse oximeters

Parameter	Point estimate (range)		Data source(s) and assumption
	Commercial tabletop device	Hand-held device	
Baseline anaesthetic-related mortality, deaths per million operations requiring general anaesthesia	467 (253–7500)	467 (253–7500)	Systematic review of anaesthetic-related mortality^14^
Anaesthetic-related deaths averted by oximetry, %	10 (3–50)	10 (3–50)	Authors’ estimates based on intermediate outcomes from systematic review of randomized control trials^22^ and observational data^14^^,^^28^^,^^29^
Discounted DALYs per death avoided^a^	15.5 (10–30)	15.5 (10–30)	Authors’ calculation based on approximation of age distribution of patients undergoing surgery^36^ and health-adjusted life expectancy by age^35^
Number of times each oximeter used per week	22 (20–30)	22 (20–30)	Assumed 80% utilization^8^ of maximum capacity of 25–30 operations per week^12^
Discounted DALYs averted per year of oximeter use	0.83	0.83	Authors’ calculation
Equivalent annual cost of oximeter, US$^b^	310	95	See Table 1
Cost per DALY averted, US$^c^	374	115	Authors’ calculation

DALY: disability-adjusted life-year; US$: United States dollars.

^a^ Based on region-specific life-tables.

^b^ From Table 1, in 2013 values.

^c^ In 2013 values.

The purchase of a hand-held oximeter for each of the 77 000 operating theatres globally that currently do not have pulse oximeters^3^ would cost about US$ 19.3 million. Using the parameters in this paper, we estimate that equipping all of these operating theatres with pulse oximeters would reduce the global burden of disease by 63 800 DALYs per year.

### Sensitivity analyses

Given the paucity of trial data and the uncertainty surrounding the effectiveness of pulse oximetry in averting anaesthetic-related mortality, we explored the sensitivity of our results to variation in the key parameters.

**Fig. 1:**
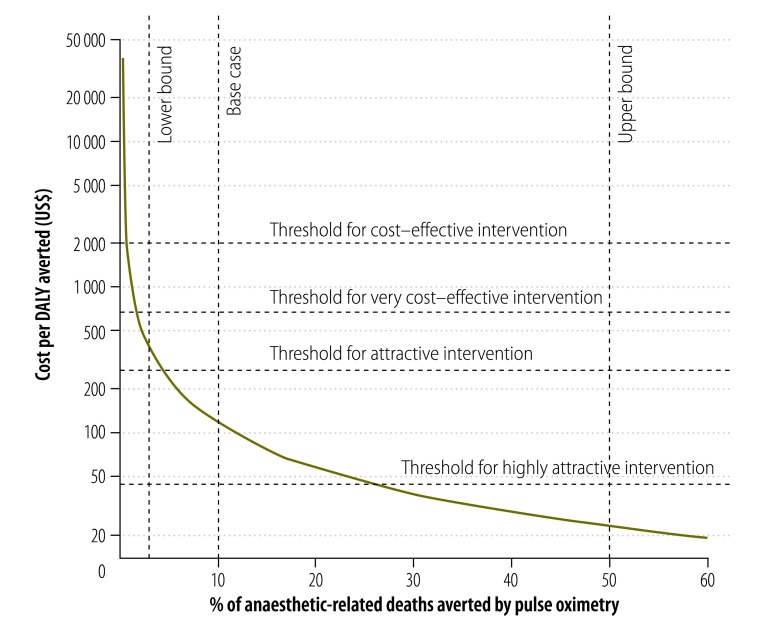
Cost–effectiveness of pulse oximetry as a function of the proportion of anaesthetic-related deaths averted

DALY: disability-adjusted life-year; GDP: gross domestic product; US$: United States dollars.

Note: The cost–effective thresholds were for the year 2013. Attractive interventions were based on the *World development report 1993.*^38^ Cost–effective interventions were based on World Health Organization guidelines, with very cost–effective and cost–effective interventions below one (US$ 677) and three (US$ 2031) times the GDP per capita for the group of low-income countries, respectively^7^^,^^39^

**Fig. 2:**
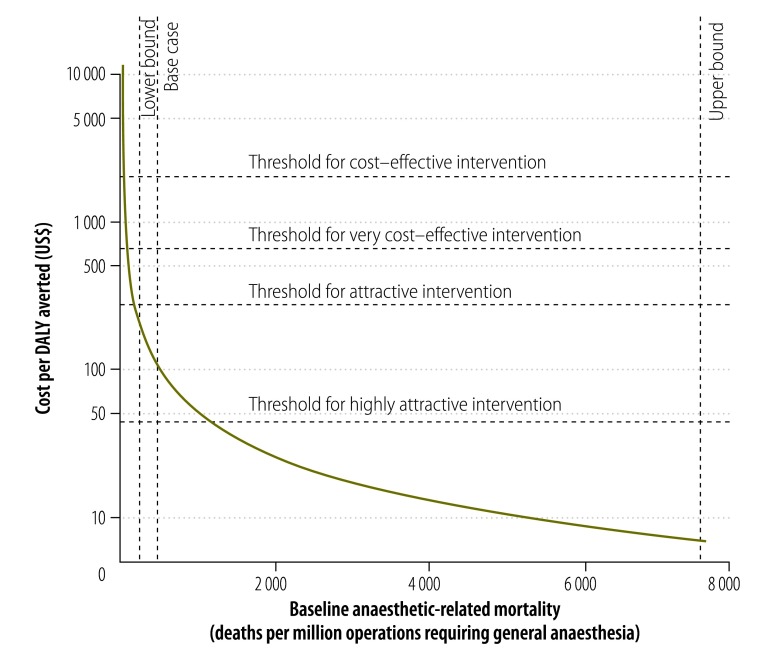
Cost–effectiveness of pulse oximetry as a function of baseline anaesthetic-related mortality

DALY: disability-adjusted life-year; GDP: gross domestic product; US$: United States dollars.

Note: The cost–effective thresholds were for the year 2013. Attractive interventions were based on the *World development report 1993.*^38^ Cost–effective interventions were based on World Health Organization guidelines, with very cost–effective and cost–effective interventions below one (US$ 677) and three (US$ 2031) times the GDP per capita for the group of low-income countries, respectively^7^^,^^39^

Fig. 1 shows the cost per DALY averted as a function of the percentage of anaesthetic-related mortality prevented by pulse oximetry. The hand-held pulse oximeter falls below the attractive threshold for 2013 from the *World development report* *1993*, if it prevents 4% of anaesthetic-related mortality. It falls below the GDP per capita of the group of low-income countries if it prevents 1.7% of anaesthetic-related mortality (0.3% of total peri-operative mortality). The wide variation seen in levels of anaesthetic-related and total peri-operative mortality between settings has an impact on the cost–effectiveness of pulse oximetry.^14^^,^^16^^,^^17^^,^^41^ Fig. 2 shows the cost of a hand-held pulse oximeter, per DALY averted, as a function of baseline anaesthetic-related mortality, assuming that pulse oximetry prevents 10% of anaesthetic-related deaths. With baseline anaesthetic-related mortalities of 253^41^ and 7500^17^ deaths per million operations requiring general anaesthesia, a hand-held pulse oximeter would have cost US$ 211 and US$ 7 per DALY averted, respectively.

The base case, lower bound and upper bound are our own estimates of the proportions of anaesthetic-related deaths that could be averted by the routine use of pulse oximetry.

The base case,^14^ lower bound^14^ and upper bound^17^ report levels of the baseline anaesthetic-related mortality that occurs – or might occur – in the absence of pulse oximetry.

**Fig. 3:**
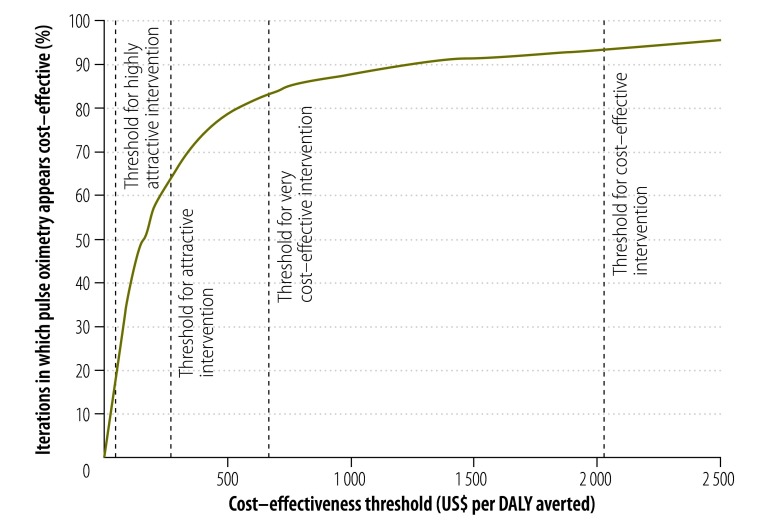
Cost–effectiveness acceptability curve for pulse oximetry

DALY: disability-adjusted life-year; GDP: gross domestic product; US$: United States dollars.

Note: The cost–effective thresholds were for the year 2013. Attractive interventions were based on the *World development report 1993.*^38^ Cost–effective interventions were based on World Health Organization guidelines, with very cost–effective and cost–effective interventions below one (US$ 677) and three (US$ 2031) times the GDP per capita for the group of low-income countries, respectively^7^^,^^39^

We conducted a basic probabilistic sensitivity analysis by carrying out 1000 iterations using the parameter distributions given in Table 3. The effectiveness of oximetry in averting death was assumed to be independent of baseline anaesthetic-related mortality. Although the median incremental cost–effectiveness was US$ 154 per DALY averted, the corresponding mean cost–effectiveness was much higher – US$ 628 – largely because of the small number of simulations with extremely low baseline mortality and effectiveness. The corresponding cost–effectiveness acceptability curve (Fig. 3) indicates that hand-held pulse oximeters are likely to be considered cost–effective – compared with no oximetry – with all but the most stringent cost–effectiveness threshold. Pulse oximetry fell under the WHO very cost–effective threshold in 83% of the simulations and under the attractive cost–effectiveness threshold from the *World development report 1993* in 62% of the simulations. The results of the probabilistic sensitivity analysis should be interpreted with caution because of our uncertainty about the relationship between identified hypoxic episodes and mortality.

**Table 3:** Parameters included in probabilistic sensitivity analysis

Parameter	Point estimate	Distribution	Distributional parameters	Data source(s)
Anaesthetic-related mortality, deaths per million operations requiring general anaesthesia	467	Log–normal	*µ* = 6.0; *σ* = 0.56	Systematic review, with variance increased to take into account higher mortality for excluded studies^14^
Proportion of anaesthetic-related deaths averted by pulse oximetry	0.1	Beta	*α* = 1; *β* = 9	Authors’ estimates based on intermediate outcomes from review of randomized control trials^22^ and observational data^14^^,^^28^^,^^29^
Annual equivalent cost of purchasing and maintaining a hand-held pulse oximeter, US$ per 1000 operations requiring general anaesthesia	83	Log–normal	*µ* = 4.4; *σ* = 0.81	Authors’ calculation

US$: United States dollars.

## Discussion

Although this study is not entirely based on hard evidence from randomized controlled trials, our results indicate that pulse oximetry is cost–effective. Obtaining parameter estimates for a decision model is often difficult. In this case, the problem was compounded by a paucity of evidence relating to use of oximetry in low-income settings and the very low frequency of the outcome of interest. To estimate the effectiveness of oximetry in averting peri-operative death, it was necessary to extrapolate from surrogate outcomes and from observational studies in high income countries. Another possibility would have been to estimate this parameter by means of a Bayesian elicitation, but the estimate would still have been an informed guess. Our approach instead was to carry out extensive sensitivity analysis. In our base case, the hand-held pulse oximeter appeared to be very cost–effective for low-income countries if it prevented just 1.7% of anaesthetic-related deaths or 0.3% of total peri-operative deaths. It is worth noting that to detect an improvement of this magnitude in total peri-operative mortality in a randomized controlled trial would require a sample size of almost 1.5 billion patients – and such a trial will never be conducted. The aim of the WHO Global Pulse Oximetry Project is to make pulse oximetry more widely available is based on best practice from high-income countries and the results of informal analysis^1^ – rather than on an explicit calculation of what oximetry would have to achieve to be cost–effective.

In this paper, we only considered deaths averted by oximetry. Our estimates of the cost–effectiveness of pulse oximetry would probably have increased if we had also considered non-fatal brain damage. Discussions with doctors working in low-income countries highlighted several additional points. First, pulse oximetry may actually reduce overall oxygen use, since flow rate can be reduced where saturation is adequate. Second, the availability of oximetry may change clinical practice. For example, only practitioners with access to oximetry may be willing to use alternatives to general anaesthesia that may be safer in some situations – e.g. spinal blocks in obstetrics. Third, there is a role for oximetry outside the operating theatre – e.g. in monitoring patients in the recovery room and mothers and neonates during vaginal delivery, and reducing oxygen use in patients with pneumonia who are tachypnoeic but well saturated.^42^^,^^43^

Our analysis considered only stand-alone tabletop and hand-held oximeters. A third type of oximeter, the fingertip oximeter, is even cheaper than the hand-held devices – with a purchase cost of US$ 30^44^ – but is designed only for spot-checks in primary care and probably has limited usefulness in operating theatres, since it lacks an audible tone that changes with oxygen saturation, an alarm to indicate desaturation and a plethysmograph display. As well as stand-alone pulse oximeters, pulse oximetry may be built into other devices – e.g. anaesthesia machines or sphygmomanometers – or combined with electrocardiography or capnography in a multivariable monitor.^45^ In practice the choice of which type of oximeter to purchase is likely to depend on a variety of setting-specific considerations. For example, in a setting with only intermittent electricity supply, a standard tabletop oximeter would be unsuitable because of its inability to function for long periods without mains electricity. The presence of combined capnography or other functionality in an expensive unit that can be used for oximetry is only valuable if the requisite expertise is present.^46^

There is a large body of literature relating to cost–effectiveness of health interventions in low-income countries.^5^^,^^7^ Much of this literature relates to evaluations of complex interventions that are of little value in specific device-procurement decisions. There is also an emerging interest in frugal innovation – i.e. the adaptation of existing medical technologies to make them more affordable and more suitable for use in low-resource settings.^6^^,^^40^^,^^47^ We hope that analysis of the type presented here – in which the types and grades of device available for a particular purpose are made explicit – could help bridge the gap between the literature on cost–effectiveness of health interventions and the literature on technical specifications for devices, allowing decision-makers to proceed beyond the prioritization of complex interventions to the selection of specific devices for different clinical settings.^46^
